# “Early Psychosis” as a mirror of biologist controversies in post-war German, Anglo-Saxon, and Soviet Psychiatry^†^

**DOI:** 10.3389/fpsyg.2013.00481

**Published:** 2013-07-29

**Authors:** Lara Rzesnitzek

**Affiliations:** Institut für Geschichte der Medizin, Campus Charité Mitte, Universitätsmedizin CharitéBerlin, Germany

**Keywords:** latent schizophrenia, sluggish schizophrenia, prodrome, vulnerability, early psychosis, subjective symptoms, basic symptoms

## Abstract

The English term “early psychosis” was coined in the 1930s to refer to feelings of irritability, loss of concentration, hypochondriac ideas, moodiness, and lassitude that were seen to precede the onset of clear-cut hallucinations and delusions. The history of thinking about “early psychosis” under names such as “latent,” “masked,” “mild,” “simple” or “sluggish” schizophrenia before World War II and afterwards on the different sides of the Wall and the Iron Curtain reveals “early psychosis” as a mirror of quite aged international biologist controversies that are still alive today and to the same extent as they are misunderstood, are influential in their implications in today's psychiatry.

The fifth revision of the Diagnostic and Statistical Manual of Mental Disorders (DSM) for the first time includes a category named “*attenuated psychosis syndrome*” as a condition for further studies (Yung et al., 2012). What had been proposed at the beginning of the revisions, however, was the introduction of “*psychosis risk syndrome*” as a new diagnosis to describe a condition with a recent onset of modest, psychotic-like symptoms with clinically relevant distress that would indicate a significantly increased risk of conversion to schizophrenia. Vigorous debates among international psychiatrists finally came to the conclusion that it might be premature to recommend a new category primarily based on future “risk” (Yung et al., 2010).

The departing point of these debates seemed to be a dissent about the meaning of “risk for schizophrenia.” It is not only the way that risk criteria differed within a “near Babylonian speech confusion” about terms as “*prodrome*,” “*early psychosis*,” “*at risk mental state*,” “*high and ultrahigh risk*” (Schultze-Lutter et al., 2011, 2012), there was—and probably still is—also a confusion about the significance of “risk for schizophrenia” tout court. Although the DSM does not claim to pinpoint disease entities, the proposed formulation of a new diagnosis, “*psychosis risk syndrome*,” did seem to implicate the existence of a disease or illness. But is a “risk for a disease” already a disease? Yes, it is, was claimed by proponents advocating the introduction of a new psychosis spectrum disorder in DSM-5 under the words “Probably at-risk, but certainly ill” (Ruhrmann et al., 2010); no, it isn't, was claimed by others. Interestingly, this up-to-date controversy that is still going on for the eleventh revision of the International Classification of Diseases (ICD) appears like a reflection of bygone biologist controversies in Post-War German, Anglo-Saxon and Soviet Psychiatry.

The English term “*early psychosis*” entered the stage of classical psychiatry in 1938, first of all with reference to “*early diagnosis*.” This new interest in diagnosing schizophrenia early was revolutionary in a time that had thought of schizophrenia as a disease process that per definition would lead into premature dementia: dementia praecox. The treatments by hypoglycaemia and convulsions that had been introduced since 1934 by Manfred Sakel and Ladislas Meduna caused to totter the concept of incurability and “brought into the foreground the necessity for early diagnosis,” as the most promising ameliorations were obtained in “early cases” (Mayer-Gross, 1938). Even conceptually quite a gap, then psychiatrists needed just a little step from the idea of *early detection* of a disease to the idea that the disease itself might have an *early phase* or even be a specific *early form* of a chronic disease: *early schizophrenia* or more general, *early psychosis* (Cameron, 1938a,b).

## *Early schizophrenia* or *prodromes* in classical european psychiatry

Subtle changes in mood and personality that gained significance retrospectively had of course always been part of the case descriptions in the classic textbooks on dementia praecox and schizophrenia. In Emil Kraepelin's description of dementia praecox in 1893, a potpourri of *initial* symptoms, especially of somatic kind, was given:

Usually the psychosis begins with symptoms of general malaise and uneasiness, headaches, ear noises, dizziness, disagreeable feelings in different parts of the body, insomnia and poor appetite. The sick persons become shy, withdrawn into themselves, downcast, anxious, stop working, express vague concerns especially with hypochondriac contents (Kraepelin, 1893, 439).

Concerning the talk about the “*early*” or “*initial*” symptoms, Eugen Bleuler felt he needed to add some words in order to prevent misunderstandings concerning the meaning of “*early symptoms*”:

When speaking of initial symptoms of schizophrenia we have to restrain us to the first symptoms that were noticed; too often we just don't know the symptoms that really appeared first.

In the corresponding footnote he went on to explain:

We do not speak of “prodromes.” We might differentiate prodromes of a seizure and inter-current signs from the full-blown seizure, if we like—prodromes of a disease, however, I am not able to imagine. What are named in this way are the first symptoms that we are not able to interpret in the right way (Bleuler, 1911, 206).

Speaking of “*prodromes*” was, however, quite common in European Psychiatry. Years before Bleuler proposed his concept of “*schizophrenia*” as a substitute for Kraepelin's “*dementia praecox*,” notions such as “*depressive prodromes*” or “*prodromal pseudoneurasthenia*” had already been discussed in the continental psychiatric literature (Pascal, 1906, 1907).

No matter what words were used, these quotations clearly show that the feelings of irritability, loss of concentration, hypochondriac ideas, headaches, moodiness, and lassitude that were seen to precede the onset of clear-cut hallucinations and delusions since the earliest descriptions of dementia praecox or schizophrenia were not conceptualized as “risk” signs for the occurrence of a disease, but they were seen as already manifesting the disease *process:* However, “what this schizophrenic process consists in, we don't know,” admitted Bleuler in 1911, even if there were clear findings of mild brain atrophy and specific histological changes in severe cases. Bleuler continued:

The question if there might be a specific brain disposition to schizophrenia and how it would manifest has still not been addressed at all (Bleuler, 1911, 376f).

## Bleuler's *latent schizophrenia*

Bleuler's favorite explication of the pathomechanism of schizophrenia was the idea of an infection or autoimmune process, which may manifest in a chronic or acute manner and may even stay *latent* over a longer period (Bleuler, 1911, 376f). Not surprisingly, *latent schizophrenia* was considered a very widespread and underdiagnosed phase of schizophrenia with fuzzy boundaries especially to *schizophrenia simplex* at first extensively described with patient examples by Otto Diem in 1903 (Diem, 1903). By separating this form of schizophrenia from *hebephrenia*, as opposed to Kraepelin, Bleuler gave *schizophrenia simplex* and *latent schizophrenia* the central exemplary position in his theory of “schizophrenia,” demonstrating his advocated dichotomy between *fundamental* symptoms (e.g., cognitive or emotional blunting) and *accessory* symptoms (e.g., hallucinations or delusions) (Bleuler, 1911, 194). Consequently, seemingly uncharacteristic symptoms such as increased distraction, forgetfulness, reduced emotional reactivity or anhedony and avolition characterized Bleulerian core schizophrenia and were therefore no risk and no prodrome: “A latent schizophrenia already is a psychosis” (Bleuler, 1917, 29).

The problem of drawing the line between character and disease was answered with resolute words:

As it is clear that many cases of schizophrenia go back into youth and as many cases impress as simply intensification of the existing character, it seems probable to me that these autistic abnormalities in character are the first symptoms themselves and not only an expression of the disposition (Bleuler, 1911, 206).

Nine years later, however, in the 3rd edition of his textbook, Bleuler used Kretschmer's term “*schizoid*” for the first time in order to admit the unresolved question of the qualitative boundaries or only quantitative differences between constitution or predisposition and disease:

As from which level of anomaly on a person should be classified solely as a “schizoid” psychopath or else as schizophrenic and mentally ill, is still not possible to define at all (Bleuler, 1920, 325).

To what extent these personality peculiarities already “are the young disease or solely expression of the predisposition,” was questioned by Bleuler especially from the point of view of genetics: of course, one must differentiate between hereditary and phenomenological visible features of schizophrenia, “Erbschizose” and “Sichtschizose,” because the hereditary features are linked to the visible ones “by a long causal chain complicated probably by the influence of some inner and external factors” (Bleuler, 1917, 31). Also “accompanying psychic predispositions, that *per se* have nothing to do with the gene of the disease, might contribute”; Bleuler here thinks of “a certain sensitivity that does not only appertain to future schizophrenics” (Bleuler, 1917, 32).

After the collapse of Nazism into “euthanasia” and World War II, West-German Psychiatry turned its back on the genetic theory of schizophrenia, much more than Bleuler—in spite of his criticism on the methodology of Ernst Rüdin's studies—would have advocated.

## The end of *classical biological* psychiatry

Already Kurt Schneider recommended his pragmatic symptomatologic classification oriented on his first (e.g., auditory hallucinations and delusions of control) and second rank symptoms, because he had capitulated in face of the indecisive results of the biologic research in schizophrenia. Bleuler's schizophrenia concept was simply but silently put aside.

With the retirement of Schneider from the chief position of the psychiatric university clinic in Heidelberg in 1955, “classical psychiatry” was said by Walter von Baeyer, his successor, “to have come to its end; the future was for existential analysis (“Daseinsanalyse”) in the sense of Heidegger, Husserl and Binswanger” (Huber, 2009, 70). In other places of European Psychiatry, the influence of psychoanalysis had already departed large parts of thinking about “early psychosis” from Bleuler's classical biological views.

Even if early schizophrenia was still in the spotlight, there was a “sort of panic” in West-German Psychiatry a year before the Wall was build: there was an anxious suspicion that everything concerning schizophrenia research had been seen and done in a wrong way (Kraemer, 1960). Almost everything of the biologic view on schizophrenia and the diagnostic methods used, was questioned as being wrong. Accordingly, in the 60s, West-German Psychiatry started to see paranoid schizophrenia and early schizophrenia in an anthropologic light and to explain them by the individual situation in the life of the concerned person: a personal *“failure on the road of life”* (Zutt and Kuhlenkampff, 1958). Similarly, as in the classic psychiatric schools situated in the west of the new Iron Curtain in Europe, the “schizophrenic person” gained center stage (Wyrsch, 1949). “Schizophrenic” characteristics were explained in the light of Heidegger's existential philosophy: “*eccentricity, crankiness, mannerism as three forms of failed existence*” (Binswanger, 1956, “Drei Formen missglückten Daseins. Verstiegenheit, Verschrobenheit, Manieriertheit”). Symptoms of beginning schizophrenia were reformulated in Heidegger's language as *“disclosure, dissolution and overwhelming as forms of loss of the existential position in life”* (Kulenkampff, 1955, “Entbergung, Entgrenzung und Überwältigung—als Weisen des Standverlustes”). At the institutional level, the separation of the departments for neurology and psychiatry in the university clinics was pressed ahead and the (West-)German Council of Science and Humanities insisted in 1960 on the implementation of professorships for psychotherapy.

Klaus Conrad, who had published a phenomenological analysis of the steps of symptom progression at the onset of schizophrenia entitled *“The Beginning Schizophrenia”* (Conrad, 1958) in 1958, criticized the diagnostic practice of the time: what traditionally had been classified as “beginning schizophrenic phase” and already had been challenged by Kretschmer's “*schizoid”*-concept into “a sensitive delusion of reference on the base of a schizoid constitution,” that would nowadays be seen, “in Frankfurt as a consequence of a deranged existential order of being, as a form of existential failure in the pursuit of life” (Conrad, 1959, 489). As a matter of fact and in contrast to Karl Leonhard who migrated to the GDR and took up the chair of the Charité-Nervenklinik in East-Berlin in 1957, Jürg Zutt and Caspar Kulenkampff abandoned the classic biological Frankfurt-Kleist-Wernicke school. But also the Heidelberg school was increasingly marked by the wish to explain the psychopathology out of individual and family psychodynamics, as is evident in the academic writing of senior physicians of the Heidelberg clinic, for example Heinz Häfner's “Existential Analytical Investigations in the Structure and Course of Psychopaths” (Häfner, 1961), or Karl-Peter Kisker's study results *“Comparative Situation Analysis of Beginning Schizophrenias and Reactive Maldevelopment in Adolescents”* (Kisker and Strötzel, 1961/62).

Not by chance, *“adolescent crisis”* or *“maturation crisis”* became the main differential diagnoses of beginning schizophrenia (Kulenkampff, 1964; Feldmann, 1967). These concepts together with the idea of “*existential failure*” reflect a way of thinking about “early psychosis” that American psychiatry had already chosen before World War II.

## Mental hygiene and the “*schizophrenic reaction*” in the DSM

Contrary to the situation in Europe, American Psychiatry generally developed independently from neurology and was decisively shaped by its founder Adolph Meyer and his psychobiological school. In opposition to the classical pre-war European view that granted psychological factors not much more than the role of unveiling the *latent biological* basic disorder, Meyer's school explained all mental diseases as “*psychological reaction types*” (Muncie, 1935). The early American favor for early detection and prevention grew exactly out of this psychological perspective: under the assumption that all mental diseases can be explained by psychological, environmental causes, it was just a logical reasoning that they might be impeded or nipped in the bud if their causes would be detected early enough and neutralized. Meyer's Mental Hygiene Movement was based on this argument (Kalinowsky, 1955).

The emigration of European psychoanalysts to America during National Socialism led to the integration of Freud's theory of intrapsychic conflict into the environmentally oriented Mental Hygiene Movement: a broadly defined psychosocial model was born that conceptualized even schizophrenia as reducible to one basic psychosocial process: Karl Menninger's *“failure of the suffering individual to adapt to his or her environment”* (Wilson, 1993, 400). Only the intensity of the trauma determined if the reaction would be of a neurotic or of a psychotic kind. Symptoms were seen in the psychodynamic light of “meaning.” As a result, the frontiers between character eccentricities and schizophrenia vanished on psychological grounds. Schizophrenia was just a more severe psychological maladjustment than other personality or neurotic abnormalities; it was no longer a genetic disease but rather a psychosocial reaction, as expressed in the revision of the Army nomenclature under the leadership of Menninger, the first DSM published in 1951, and its revealing term: “*schizophrenic reaction*.”

The psychodynamic or even psychoanalytic interpretation of *early* schizophrenia was already evident in Harry Stack Sullivan's lecture entitled *“The onset of schizophrenia,”* held on the occasion of the joint meeting of the American Psychiatric Association (APA) and the American Psychopathological Association in 1926 (Sullivan, 1927), as it is in the famous article on the *“Diagnostic evaluation of early schizophrenia”* written by Phillip Polatin and Paul Hoch in 1947 (Polatin and Hoch, 1947). The introduction of the term “*ambulatory schizophrenia*” by Gregory Zilboorg in 1941, *“pseudoneurotic schizophrenia”* by Hoch and Polatin in 1949 and the interpretation of *“Borderline States'* by Robert Knight in 1953, continued to foster this psychodynamic view on *early* and *mild* psychosis (Zilboorg, 1941; Hoch and Polatin, 1949; Knight, 1953).

Granted, three of the first articles published in English on early schizophrenia still had a classical medical model of the condition, but the articles were written by a German psychiatrist who had immigrated to London (Mayer-Gross, 1938) and by a Scottish psychiatrist who trained under the successor of Bleuler at the famous Burghoelzi Clinic in Switzerland during publication year (Cameron, 1938a,b). Even if classical views were still published in Anglo-Saxon psychiatry, their impact on thinking about “early psychosis” was almost non-existent in the years that followed World War II—just as the results of the clinical study “The Genetics of Schizophrenia” of another German refugee from National Socialism (Kallmann, 1938).

During the 1960s, the view of mental disorders as non-biological psychosocial problems became the source of anti-psychiatric arguments: “if conceived of psychosocially, psychiatric illness is not the province of medicine because psychiatric problems are not truly medical, but social, political, and legal” (Wilson, 1993, 402); mental illness was a myth and psychiatric labels arbitrary designations (Szaz, 1961). The revision of the DSM, published in 1968 by the APA as *DSM-II*, consequently dropped the term “*reaction*” even if psychodynamic views largely prevailed besides a re-appropriation of classical concepts (American Psychiatric Association, 1968). Orienting itself on the 8th revision of the ICD that listed—in classical Bleulerian tradition—as subtypes of schizophrenia “*Schizophrenia simplex*” and “*latent schizophrenia*” (World Health Organisation, 1965), the APA also consented on a “*simple type*” and a “*latent type*” of schizophrenia. In explaining “*latent schizophrenia*,” however, it was added that—among “incipient” and “pre-psychotic”—“pseudo-neurotic, pseudo-psychopathic, or borderline-schizophrenia are categorized here”—which clearly were of psychodynamic origin (American Psychiatric Association, 1968).

## *Sluggish schizophrenia* in soviet psychiatry

Soviet Psychiatry strictly rejected western anthropological interpretations of mental illness denunciating these views “as a sign of a severe crisis in capitalistic countries' psychiatry” (Sternberg, 1964).

Characteristic for Soviet psychiatry was not only its clear biological orientation, but also especially its preoccupation with Bleuler's “latent schizophrenia.” Clinical research started as early as 1924 at the *Moscow Institute for Neuropsychiatric Prophylaxis* and centered on the questions of “*mild*,” “*attenuated*” or “*masked*” schizophrenia. However, Bleuler was criticized for using the word “*latent*” in a context where schizophrenia was already manifest, but in a mild, non-psychotic form, just as he himself had very well tried to explain, but was easily misread by the unclear signification of the word “*latent.*” As a consequence, *mild* or *sluggish* schizophrenia was assumed to consist of a sort of *attenuated organic*, perhaps toxic, *process with slow progression* (Kameneva, 1935). The director of the Institute for Neuropsychiatric Prophylaxis of the time, L. M. Rosenstein, himself pointed out that the elaboration of the concept “*sluggish schizophrenia*” was conditioned by the politically enforced re-structuring of the medical psychiatric facilities with closure of private consultations and a concentration on polyclinic centers. “The moments that mostly determine the development of scientific categories of our discipline are the current historically-given forms of our psychiatric practice,” wrote Rosenstein in his report about the new achievements concerning “early psychosis” since the foundation of the Soviet Union (SU) in 1922. The most recent form of psychiatric *practice*, “namely the set-up of psychiatric welfare units called ‘dispensaries’ ” where “psychiatrists are facing a material, that usually counts as ‘healthy’ or ‘nervous’ and will have to do prophylactic work on it,” is feeding back on the theoretical concepts (Rosenstein, 1933, 299f). The parallel of the *dispensaries* to the institutional development inside the American Mental Hygiene Movement is quite interesting due to the different if not opposing theoretical foundations. In Europe, attention had been paid to the mild forms of schizophrenia until the end of World War II after the classic description of “Heboidophrenia” by Karl Ludwig Kahlbaum and “Dementia simplex” by Diem, especially in the context of the growing acceptance of another psychiatric practice: *psychotherapy* (Kronfeld, 1928; Wyrsch, 1945).

The focus of clinical interest on bland, mild or sluggish schizophrenias was to shape the whole Soviet theory of schizophrenia that was seen as a life-long process of a genetically determined disease (Sternberg, 1973). Classification remained oriented on the course or progressive evolution of symptoms seen in the “unitary psychosis layer model” formulated by Andrej Sneshnewski (Piatnitski et al., 1998).

## Gerd huber and gisela gross as advocates of the classical views in west germany

Few West-German psychiatrists have been invited for lectures in the German Democratic Republic (GDR). Thanks to an invitation of the (East-)Berlin Society for Psychiatry and Neurology at the Humboldt-University, Gerd Huber was among them to present his pneumencephalographic studies in schizophrenia in the year 1958 (Dietrich, 1958). The university psychiatric clinic of Heidelberg with its growing focus on anthropological thinking had just generously allowed Huber to finish his compilation of pneumencephalographies taken at the onset of schizophrenia that he had started in 1950 in order to correlate psychopathological symptoms with localized brain atrophy (Huber, 1957a). The description of “*coenaesthetic schizophrenia*” that Huber published in the same year (Huber, 1957b), was sparely appreciated in West-German Psychiatry, but was received with emphatic approval in the SU and GDR. It is no surprise then that the clinical “differentiation of hypochondriac syndromes,” was seen “as currently one of the most difficult and urging psychiatric problems” in Soviet psychiatry. Consequently, Huber's work was applauded as an important contribution to the organic base of “*hypochondriac schizophrenia*” as already described by the Russian psychiatrist G. A. Rothstein (Sternberg, 1964).

As the leading physician of the psychiatric outpatient department of the Heidelberg university psychiatry, Huber had been able to conduct his barely connived follow-up examinations until 1962 for the construction of his “Heidelberg Checklist of Basic Symptoms” (Huber, 1962) that is nowadays well known under the name “Bonn Scale for the Assessment of Basic Symptoms” (*BSAB*) and is used as *“an instrument for the assessment of schizophrenia proneness”* (Gross et al., 1987; Klosterkötter et al., 1997).

“Barely connived” was Huber's psychopathologic assessment of subjective complaints of patients with early schizophrenia because of the biological idea on which they were founded. The reason why Huber was interested in subjective experience and feelings was solely because he thought that they would shed light on the organic base of schizophrenia. The *subjective symptoms in early psychosis* were credited to lead directly to the *biologic* “*fundamental”—“primary”—or “basic”—symptoms*.

The subjective experience of subtle cognitive deficits and changed self-feeling were originally identified by Huber as “*pure defect*” in chronic schizophrenia after the psychotic symptoms had disappeared, but then recognized in the prodromal phase. The notion of “*basic symptoms*” was used to make clear that these subtle feelings are the core symptoms, the “most primary symptoms” of schizophrenia in the sense of their direct organic origin (Huber, 1966). Even though some of the contents of paranoid ideation in full-blown schizophrenia might be explicable by the individual personal situation of the affected person, what is seen in early psychosis is the direct expression of the organic origin of schizophrenia according to Huber and not analyzable in the frame of existential psychiatry. Likewise, Klaus Conrad thought it was possible to extract an analysis of the different stages of “beginning schizophrenia” out of the examination of uniformed soldiers realized during World War II: the question was not about individual conflicts and situations but about *the neuropsychological laws of symptom progression* at the onset of schizophrenia (Conrad, 1958). Evidence for the neurobiological determination of the different stages of the changing experience in early schizophrenia would also have been searched for by Conrad with biological means, just as he already had tried to find the genetics of epilepsy or schizoid constitution, if he had not died in the year of the construction of the wall before assuming the directory of the Max Planck Institute for Psychiatry in Munich.

Huber, his lifelong co-worker Gisela Gross and sympathizing psychologists nevertheless arrived at pinpointing “basic” symptoms even in *“the failure state of latent schizophrenia”* (Blankenburg, 1968), a denomination that might associate an anthropological psychodynamic account in Menninger's tradition as “failure to adapt to personal life challenges.” On the contrary, the “*juvenile-asthenic failure-syndromes,”* as Huber called “*the failure states of early psychosis*,” were traced back to an organic base (Glatzel and Huber, 1968). This way, a seemingly uncharacteristic symptomatology was conceptualized as “abortive, latent or masked schizophrenia” (Gross et al., 1982). The number of colleagues who sympathized with this view in West Germany might be counted on one hand relying on the BRD-psychiatrists who followed Huber's invitation to the *Weißenauer Symposien*. The first symposium, which still took place at the psychiatric hospital “Weißenau” in 1971 (at this time under the directory of Huber before he finally found refuge in Bonn and the Weißenauer Symposion with him), was not accidentally dedicated to the etiology of schizophrenia, and defined a clear biologic direction of future research (Huber, 1971). The extent to which this alignment was rejected as outdated and obsolete in the 70s in West-Germany is mirrored in the blatant opposition of the audience that Huber encountered during his lecture on schizophrenia on the occasion of his application for the directory of the Heidelberg psychiatric university clinic in 1972: the audience wove a banner with the words: “Huber, evil excrescence of bourgeois psychiatry” (Huber, 1996, 237).

## The role of psychology

Perhaps surprisingly, it was the practice of psychotherapy of schizophrenia that finally led to a revival of the medical model of “early psychosis” thanks to a newly flourishing branch of psychology called “experimental psychology.”

Clinical psychologists with an originally psychoanalytic training began to recognize that cognitive deficits of patients with schizophrenia impeded psychotherapy on large grounds. This psychotherapeutic approach finally paved the way to a clinical research in psychology that tried to understand the mechanisms of the observed cognitive deficits in schizophrenia by applying the techniques of experimental psychology (Chapman et al., 1959). One of the very first programs of this kind was situated in Glasgow and headed by Arthur McGie, the Principal Psychologist at Royal DundeeLiff Hospital and honorary professor at the Department of Psychiatry at St Andrews University. As early as 1961, McGhie and a young psychiatrist, named James Chapman, published their observations on specific *“disorders of attention and perception in early schizophrenia”* (McGhie and Chapman, 1961). Chapman gained his MD with a thesis entitled *“On the early diagnosis of schizophrenia”* in 1964 and his summary publication of his results in 1966 as *“The early symptoms of schizophrenia”* became the starting point for other psychologists all over the world to reconsider early psychosis on empirical and finally biological grounds as shown by the example of the German psychologist Lilo Süllwold and her *Frankfurt Complaint Questionnaire* that is the instrument most widely used in Europe for assessing subjective experience in schizophrenia (Chapman, 1964, 1966; Süllwold, 1977).

Initially employed for a research program about the family psychodynamics of pre-schizophrenic adolescents at the psychiatric university clinic in Heidelberg, she started to collect complaints of subjective cognitive deficits in these young patients. Already in her first presentations on the occasion of the *Weißenauer Symposien* in 1971 and 1973, Süllwold explicitly combined her phenomenological approach—for which she cites McGhie and Chapman—with a biological interpretation of the observed malfunctioning (Süllwold, 1971, 1973). The gradually developing *Frankfurt Complaint Questionnaire* aimed to enable a reliable early differential diagnosis of pre-psychotic schizophrenia in contrast to neurotic troubles (Süllwold, 1973), even if Süllwold, just as McGhie und Chapman, was not just interested in early diagnosis, but finally also in a reapplication of the findings for cognitive behavioral psychotherapy (Chapman and McGhie, 1963).

Interestingly, the Anglo-Saxon results of primary attention and perceptual deficits in schizophrenia matched with the Soviet experimental schizophrenia research of Poljakow (Poljakow, 1971) for example. In the first publication of her *Frankfurt Complaint Questionnaire*, Süllwold referred to Poljakow the same way she had already pointed out in her presentation of the very beginnings of her complaint list, that the experimental research on schizophrenia conducted by Anglo-Saxon clinical psychology, eventually accomplishes Kraeplin's demand and so tied in with the tradition of classic psychiatry (Süllwold, 1971, 37; Süllwold, 1977).

Actually, there was a remarkable intertwining on the subject of experimental psychological research on the perceptual and cognitive deficits in schizophrenia across the Iron Curtain. Frank Fish for example, a Scottish psychiatrist, summarized the newly developed neuropsychological test methods for his colleagues in West-Germany (Fish, 1966) and was invited, on the other side of the Wall, by the East-German psychiatric journal, to present his own neuropsychological testing results in schizophrenia (Fish, 1965). Equally, papers about experimental psychology in their significance for the biological theory of schizophrenia and their basic symptoms were welcome in the East even if written by west-psychologists (Plaum, 1978). The transfer was clearly not achieved by psychology as such, but by psychology as a servant of biological psychiatry.

Especially the question of *subjective symptoms of beginning schizophrenia*, that Conrad had initially called to mind after World War II, as well as their neuropsychological, neurobiological explanation by Huber and Gross (Gross, 1969) and Süllwold (Süllwold, 1977) in West-Germany, McGhie and Chapman from the UK (Chapman, 1966), Dudek from Canada (Dudek, 1969), and Freedman and Chapman, USA (Freedman and Chapman, 1973), started to form a bridge over the Iron Curtain. Opposing the American psychiatric tradition of Menninger with its psychodynamic view on schizophrenia, Fish outlined his neurophysiologic theory via Conrad's phase-model of beginning schizophrenia (Fish, 1961).

The mission of psychology in this context (McGhie, Süllwold, Chapman and Freedman all of them were psychologists) was couched in the clearest possible terms by the American psychologist Paul Meehl in his lecture addressed to the American Psychological Association in 1962: “in the near future” psychology with its new experimental techniques will help “to establish that schizophrenia, while its content is learned, is fundamentally a neurological disease of genetic origin” (Meehl, 1962).

Meehl's taking side with genetics and his concept of “*schizotaxia*” as genetic foundation of the “*schizotype*” character, the last being only the compensated form of schizophrenia, as in clinically compensated cardiac or kidney disease, did not appeal much to the large parts of psychiatry and psychology that still held on to psychodynamics up to the end of the 70s (Meehl, 1989). The American psychiatrist Joseph Zubin together with the psychologist Bonnie Spring were to have greater success in bringing together the warring parties by integrating all available psychological, biological and social aspects into a recycled concept of “*vulnerability*” (Zubin and Spring, 1977). By no longer defining “vulnerability” as “*causa interna*” but as “the empirical *probability* that an individual will experience an episode” of schizophrenia, Zubin and Spring admitted any possibilities of how this inclination comes about: it may be of genetic origin, it may be caused by acquired etiological factors such as perinatal complications, substance abuse but also “just” by family stress. However, “vulnerability” is generally seen as meaning more than “probability” or “risk” because a causal claim is implicitly made concerning the enumerated factors.

## *Latent schizophrenia* and *prodromal symptoms* after 1980

The overabundant labeling of schizophrenia due to psychodynamic presuppositions in American Psychiatry and due to its concept of “soft” or “sluggish schizophrenia” in Soviet Psychiatry had meanwhile come to light in 1973 with the publication of the first results of the *International Pilot Study of Schizophrenia* that was conducted by the World Health Organization since end of the 60s (World Health Organisation, 1973). With the desire to enhance diagnostic reliability and thus re-open possibilities for meaningful research, the APA decided on a 3rd revision of the DSM that was to be a non-theoretical purely descriptive manual with emphasis on the assessment of easily observable symptoms for objective measurement (American Psychiatric Association, 1980). Consequently, *“simple”* and *“latent” schizophrenia* disappeared; diagnosis of schizophrenia completely oriented itself toward Schneiderian first as well as second rank symptoms. On the other hand, non-psychotic, schizophrenia-like disorders were classified as *“schizoid”* or *“schizotype personality disorders.”*

However, the symptoms of simple and latent schizophrenia also found refuge under another label: the list of *prodromal* symptoms that enumerated eight mostly behavioral, observable, so-called “*negative symptoms*”: “1, social isolation; 2, marked impairment in role functioning; 3, markedly peculiar behavior; 4, marked impairment in personal hygiene and grooming; 5, blunted, flat, or inappropriate affect; 6, digressive, vague, overelaborate, circumstantial, or metaphorical speech; 7, odd or bizarre ideation, or magical thinking and 8, unusual perceptual experiences.” That list was added with “9, loss of energy” in the DSM-III-R in 1987.

In the SU, the classic concept of “*early psychosis*” in the sense of “mild” or “latent” schizophrenia lived on without any challenge due to the application of a self made classification system for mental disorders completely independent of DSM and ICD. Due to this system, developed at the Moscow Psychiatric Institute by Sneshnewski, a wide concept of “schizophrenia” remained in place that also encompassed the non-psychotic forms. The study of these “mild” forms of schizophrenia had remained a core theme of Soviet psychiatric research until end of the 80s. Many subtypes of mild schizophrenia have been differentiated, among them “*simplex-schizophrenia*,” “*hypochondriac schizophrenia*,” “*hysteriform schizophrenia*,” forms with predominant *depersonalization* or affective symptoms, “*anancastic schizophrenia*” or “*psychopathic like schizophrenia in childhood*” (Sneshnewski, 1977; Piatnitski et al., 1998).

These biological concepts of schizophrenia united psychiatrists across the Iron Curtain in such a way that enabled international symposia in the SU, as the “Biological and Genetical Aspects of Schizophrenia” symposium in 1973 that was jointly organized by the World Psychiatric Association and the Moscow Academy for Medical Sciences.

Nevertheless, in the course of the political misuse of psychiatry in the SU, the Soviet concept of “*sluggish schizophrenia*” was criticized concerning its possible misuse for political reasons (Merskey and Shafran, 1986).

Shortly after the fall of the Wall and before the end of the SU, two symposia took place in 1990 mirroring the lasting controversial position of the “early psychosis” concept: the presentation of the first prospective study on early schizophrenia on the occasion of the 8th Weißenauer Symposion in March 1990 and the Symposium *“Symptoms of schizophrenia that are not criteria of DSM”* at Annual Meeting of the APA in New York, May 1990.

The first prospective study on early diagnosis of schizophrenia has been initiated by Huber and Gross in 1970, was founded by the West-German Ministry for Research and Technology and was later on continued by Joachim Klosterkötter under the name of “*CologneEarlyRecognition-Study*” *(CER)* (Gross et al., 1992; Klosterkötter et al., 2001). At the 8th Weißenauer Symposion, the discussion that followed the two lectures presenting the very first results of the *“Basic-symptom oriented diagnostic of schizophrenic vulnerability”* (Gross et al., 1990; Klosterkötter et al., 1990) became a crossfire: due to the fact that the basic symptoms that served the description of the “prodrome” now sailed under the flag of Zubin's “vulnerability,” the question arose if vulnerability really always already is a pathology and sign of a disease. If “vulnerability” equaled “prodrome,” wouldn't this mean that the prodrome—and with it the initial phase of schizophrenia, would be present from birth on, if one credited genetics or perinatal trauma with a role in vulnerability? Accordingly, would the term “vulnerability” equal “*compensated*” or “*latent schizophrenia*”? Would “vulnerability for schizophrenia” already *be* schizophrenia? The oscillation of the conceptualization of the basic symptoms between *state* or *trait* markers was of course not entirely innocent for this ambiguity. Moreover, anticipating the objection that the basic symptoms that were used for the diagnosis of schizophrenic vulnerability had not proved to be specific for schizophrenia, Gisela Gross frankly declared that there would not exist any specific psychopathological phenomena at all in psychiatry—and thus made a comment in the direction of an unspecific vulnerability in the sense of a strong “unitary psychosis” model of mental disorder comparable to the Russian “layer-model,” yet continued by arguing that the basic symptoms would not exist in personality or neurotic disorders. Thus she corrected herself to a sort of weak “unitary psychosis” model of affective and schizophrenia disorders. However, as a matter of fact, the work of the Bonn School on early diagnosis has been understood as if there would be a schizophrenia specific cognitive vulnerability that could be identified by the subtle psychopathological examination via the “Bonn Scale” and would enable early detection and early treatment (Klosterkötter et al., 2001). In any case, current formulations as “Diagnosing schizophrenia in the initial prodromal phase” make just too clear that “prodrome” is seen in a classical Bleulerian perspective as the initial state of schizophrenia and not as “risk/vulnerability” for schizophrenia (Klosterkötter et al., 2001).

At the APA symposium in 1990, Huber and Gross argued for their classic view on early psychosis, basic symptoms and prodromes. Nevertheless, the list of prodromal symptoms was dropped for the DSM-IV in 1994: without any alternative.

Even if the ICD-10 still knows of *schizophrenia simplex* (World Health Organisation, 1992), no criteria are given neither in the DSM-IV nor the ICD-10 to diagnose “prodromes” of schizophrenia or “latent schizophrenia”. Under the strong promotion of the professional descendants of Huber and Gross, however, the DSM-5 has now introduced “*attenuated psychotic syndrome*” as a research category, which may well be seen—just as the originally proposed “*psychosis risk syndrome*”—as standing in the tradition of “*early psychosis*” or “*latent schizophrenia*” that the article has recalled.

### Conflict of interest statement

The author declares that the research was conducted in the absence of any commercial or financial relationships that could be construed as a potential conflict of interest.
